# Influence of Alginate Properties and Calcium Chloride Concentration on Alginate Bead Reticulation and Size: A Phenomenological Approach

**DOI:** 10.3390/polym15204163

**Published:** 2023-10-20

**Authors:** Chanez Bennacef, Stéphane Desobry, Jordane Jasniewski, Sébastien Leclerc, Laurent Probst, Sylvie Desobry-Banon

**Affiliations:** 1 Université de Lorraine, Laboratoire d’Ingénierie des Biomolécules (LIBio), ENSAIA, 54000 Nancy, France; chanez.bennacef@univ-lorraine.fr (C.B.); stephane.desobry@univ-lorraine.fr (S.D.); jordane.jasniewski@univ-lorraine.fr (J.J.); 2 Cookal Company, 19 Avenue de la Meurthe, 54320 Maxéville, France; laurent.probst@cookal.fr; 3 Université de Lorraine, CNRS, LEMTA, Faculty of Science and Technology, 54000 Nancy, France; sebastien.leclerc@univ-lorraine.fr

**Keywords:** alginate, ionic gelation, modeling, phenomenological model, response surface method, alginate bead size, molecular weight, M/G ratio

## Abstract

Two types of alginates, AlgLF and AlgP, were used in this study to produce alginate beads by electro-vibratory extrusion. AlgLF and AlgP exhibited different Mannuronate/Guluronate (M/G) ratios and molecular weights as measured by NMR and SEC-MALS. The calcium chloride concentration was found to have the greatest effect on bead size. Higher concentrations resulted in smaller beads. AlgLF with a higher molecular weight and a lower proportion of G blocks showed smaller beads. For both alginates, the bead size was also influenced by the flow rate and vibration frequency. Alginate solution aging showed a minimal effect. Alginate reticulation was modeled using a mathematical equation. The study provides insights for the optimization of alginate-based materials in different applications by shedding light on the main factors influencing bead size. The importance of the molecular weight, M/G ratio and calcium ion concentration in the gelling process is highlighted, providing opportunities for the tailoring of alginate materials through a phenomenological model.

## 1. Introduction

Encapsulation technology has gained widespread use, particularly for the stabilization and controlled delivery of compounds of interest, including active food ingredients and probiotics. Depending on the encapsulation purpose and core material’s nature, various encapsulation methods can be employed [1,2,3]. Although the encapsulation technology adds costs to the final product, it offers numerous benefits to the food industry. Extrusion is a conventional and cost-effective method that can be carried out under mild conditions, resulting in the minimal loss of entrapped bioactive components [4]. Extrusion technology consists of the injection of a polymeric solution (e.g., alginate) containing active ingredients through a nozzle into an ionic gelling bath containing typically calcium ions [5]. Several techniques are derived from dripping extrusion for bead production. They usually differ in droplet formation and the separation step [6]. In the present paper, electro-vibrating extrusion was chosen to produce beads.

The use of a polymer matrix in encapsulation technology provides protection for active components against external factors such as oxygen, light, humidity and heat, thus improving the stability and functionality during processing and storage [7]. In the food industry, this technology also helps to mask unwanted flavors and colors that may result from the addition of bioactive compounds such as polyphenols and fatty acids, without altering the food’s sensory quality [8].

Among the numerous natural polymers used for encapsulation, alginate is one of the most used in bead production, being non-toxic, biocompatible, biodegradable and Generally Recognized As Safe (GRAS) [9]. Alginate is extracted from brown marine algae and some bacteria, such as *Pseudomonas aeruginosa*. It is composed of β-D-mannuronic acid (M unit) and α-L-guluronic acid (G unit) chains, forming MM, GG and MG sequences [10,11]. The percentage of M and G units varies depending on the species from which the polymer is extracted and the treatment that it has undergone [12]. Different M/G ratios and molecular weights result in alginates with different properties when crosslinked with calcium ions [13], usually represented as the “egg-box model” [14].

Alginates that are rich in G have a greater tendency to undergo ionic gelation. As a result, G-rich alginates tend to offer more ordered and stiffer hydrogels compared to other types of alginates, thus producing a hydrogel network that maintains its integrity for longer periods of time; however, these gels present larger pores [15]. On the other hand, alginates with a higher M content create an elastic and softer hydrogel and less permeable gel matrices.

In this study, two different alginates presenting different molecular weights, mannuronate and guluronate content and distribution were used to produce alginate beads. An experimental design was used to examine the impact of the process parameters on the alginate’s gelation and bead size.

Based on the experimental design results (response surface), a phenomenological model was proposed to predict the size variation of the alginate beads. A dimensionless parameter, C, allowed us to highlight the effect of the calcium chloride concentration (in the gelling bath) on the alginate network structure (swelling, cross-linking, contraction and erosion). This phenomenological approach was applied to both types of alginates.

## 2. Materials and Methods

### 2.1. Materials

High-viscosity sodium alginate was purchased from Louis-François (AlgLF) (Croissy-Beaubourg, Collegien, France), sodium alginate Protanal LF200 (AlgP) was purchased from FMC BioPolymers (Lyon, France) and calcium chloride ≥94% was purchased from VWR (VWR Chemicals^®^, Briare, France). NaNO_3_, EDTA and bovine serum albumin were purchased from Sigma-Aldrich (Saint Quentin Fallavier, France). Finally, PEO24k and Dextran standards were purchased from Viscotek PolyCal Standards (Malvern Panalytical, Palaiseau, France).

### 2.2. Alginate Characterization

#### 2.2.1. Molecular Weight with Size-Exclusion Chromatography (SEC) Coupled to Multi-Detectors

SEC experiments were performed with a HPLC pump (LC10AD, Shimadzu, Noisiel, France) coupled to an autosampler (Autosampler VE 2001, Malvern Panalytical, Palaiseau, France) and a multi-detector system recording UV, light scattering (RALS/LALS) intrinsic viscosity and refractive index signals (Viscotek TDA305, Malvern Panalytical, Palaiseau, France). One SEC column (A6000M, 10 or 13 μm, 8 mm ID × 300 mm, void volume ~6 mL, total volume ~12.5 mL, Malvern Panalytical, Palaiseau, France) was equipped with a post-column nylon filter (0.22 µm). The column was equilibrated with NaNO_3_ 0.15 M, NaN_3_ 0.02% and EDTA 0.01 M in ultrapure water. The flowrate was 0.7 mL·min^−1^ and the temperature was 30 °C. Data were processed with the Omnisec software (v5.12, Malvern Panalytical, Palaiseau, France). The calibration procedure was performed with bovine serum albumin, and cross-validations were performed with the PEO24k and Dextran standards. The refractometer was used as the concentration detector and the refractive index increment value (dn/dc) used to determine the molecular weight (Mw) was 0.150 mL/g [16]. Alginate samples were solubilized in the aforementioned buffer at 2 g·L^−1^ and filtered through a 0.22 µm PES filter shortly before injection (injection volume: 100 µL).

#### 2.2.2. M/G Ratio with Nuclear Magnetic Resonance (NMR) Spectroscopy

One of the most reliable methods in determining alginates’ composition and structure is ^1^H NMR spectroscopy. The functionality of this application relies on the ability of a water-soluble alginate to bind cations and form a water-insoluble gel. The gelling behavior is entirely determined by the structure of the alginate’s homopolymer blocks. Information about the uronic acid composition of the alginate can be obtained by analyzing both the positions and relative areas of signals in the anomeric zone. This information helps to calculate the mannuronic acid to guluronic acid (M/G) ratio as well as the M and G unit distribution in the polymer chain. The physical properties of alginates are largely determined by the proportion of the three types of blocks, with the GG sequences being particularly important. Alginates with higher content of MM blocks result in softer gels, while those with more GG blocks lead to stiffer gels.

Prior to ^1^H liquid-state NMR analysis, alginate samples were partially hydrolyzed by following a two-step mild acid hydrolysis procedure to reduce their molecular weight. Here, 500 mg of alginate added to 5 mL of 96% ethanol was dissolved in 50 mL of HCl, the pH was adjusted to 5.0 with NaOH, and the solution was then heated at 100 °C for 10 min for hydrolysis. The solution was then cooled down to room temperature before adjusting the pH to 3.0 by HCl addition, and then reheated at 100 °C for 20 min. After cooling, NaOH was added to neutralize (pH 7) the solution. The alginate was then precipitated with 96% ethanol. The precipitate was collected after centrifugation and dried overnight at 70 °C. D_2_O was used to dissolve the hydrolyzed alginate (100 mg·mL^−1^). This solution was filtered using a 0.45 µm syringe filter and then introduced into an NMR tube for analysis.

NMR measurements were performed on a Bruker Avance III device (Bruker, Wissembourg, France) operating at a 400 MHz frequency and equipped with a broadband inverse detection probe. Experiments were conducted at 360 °K. Spectra were acquired with an inversion recovery procedure in order to remove the water peak. The inversion time was 2.3 s. The M/G ratios were calculated from the ^1^H NMR signal area [17].

### 2.3. Preparation of Alginate Solution

The AlgLF (or AlgP) solution was prepared by dissolving sodium alginate powder (0.8% *w*/*w*) in distilled water and kept under stirring for 3 h until complete dissolution; it was then stored at room temperature for 24 to 72 h before spherification. Alginate solutions were transferred separately into 50 mL syringes for bead production.

### 2.4. Preparation of Alginate Beads

Alginate beads were prepared using a B-395 Pro Encapsulator (Buchi, Buchegg, Switzerland) equipped with a coaxial nozzle (750 µm and 900 µm for internal and external nozzle, respectively). The bead production protocol was adapted to the available equipment to produce solid beads by injecting the same alginate solution into the internal and external nozzles simultaneously. The protocol corresponded to a simple extrusion process. Accordingly, alginate beads were produced following a design of experiments.

**Figure 1:**
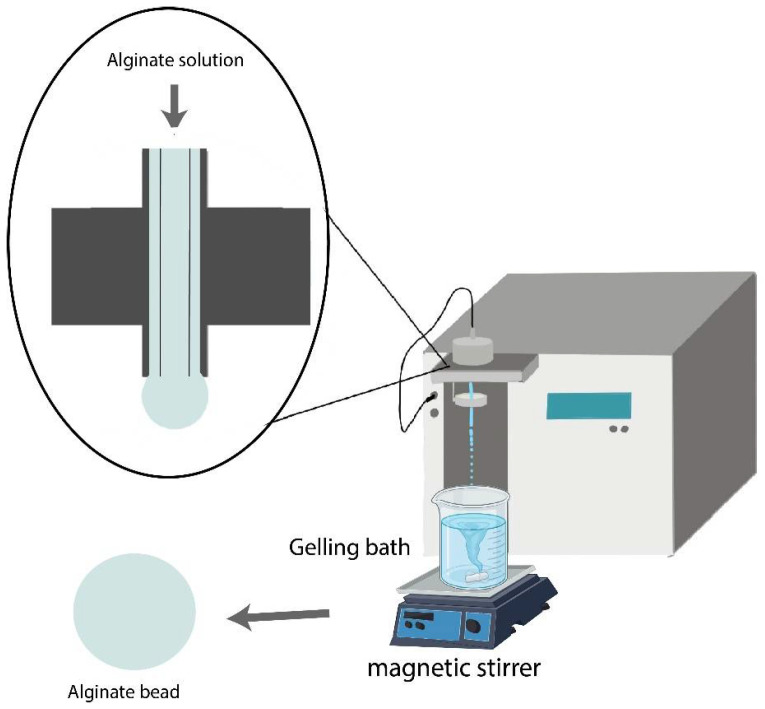
Alginate bead process used for the two types of alginate.

AlgLF bead and AlgP bead batches were produced separately. The bead production protocol is illustrated in Figure 1. Various vibration frequencies and a 2000 V electric charge were applied to the annular jet to break it into droplets.

Alginate beads were maintained for one hour in the calcium chloride solution under stirring at 400 rpm, using a 2 cm magnetic bar to ensure the complete reticulation of individual beads. They were then rinsed with distilled water and stored in distilled water at ambient temperature for analysis.

### 2.5. Bead Size Measurement

To determine the bead size, three beads from each sample were examined under an Olympus light microscope fitted with a CMOS 5 MPixels camera. To ensure accuracy, the bead area (surface) was chosen as the most appropriate measurement for the size. The Toupview 3.7 software^®^ was used for image capture and size measurements.

### 2.6. Experimental Design

An experimental design was used to evaluate the effects of five selected variables on the alginate beads’ size: the alginate’s internal and external rates, the frequency, the log_10_ of the calcium chloride concentration and the alginate solution’s age. The experimental variables are detailed in Table 1.

The Doehlert matrix was chosen in the experimental design for its flexibility and the uniform distribution of experimental points. Different levels of priority were assigned to each variable in order to emphasize the collection of data on the strongest effects. For this, calcium chloride’s effect was studied as log_10_ [CaCl_2_] to enlarge the studied concentration domain and focus on the lowest concentration, as they appeared to be crucial for alginate reticulation. Calcium concentrations corresponding to log_10_ [CaCl_2_] are displayed in Table 2.

The NEMRODW software (version 2017) was used to determine the sequence and number of experiments. The total number of experiments in the Doehlert design was determined based on the formula N ≥ K2 +K + cp, where K represented the number of variables studied, and cp represented the number of replicates at the center point. In this study, a total of 34 experiments, including three replicates at the center point, were conducted. The output data (responses) were the capsule sizes (Y), for which an empirical model was developed to predict the response function. The model showed linear, interaction and quadratic effects involving the five independent variables, as defined by the following Equation (1):

(1) $$Y=b_{0\;}+\overset{k}{\underset{i=1}{\sum }}b_{i}x_{i\;}+\overset{k}{\underset{i=1}{\sum }}b_{ii}x_{i}^{2}+\overset{k-1}{\underset{i=1}{\sum }}\overset{k}{\underset{j=i+1}{\sum }}b_{ij}x_{i}x_{j}$$

where *Y* was the studied dependent response, with *i* and *j* varying from 1 to 5 (number of factors studied). *b_0_* and *b_i_* were, respectively, a constant term and regression coefficient for linear effects; *b_ii_* were regression indices for squared effects; and *b_ij_* were regression indices for interaction effects. *x_j_* and *x_j_* were the coded experimental levels of the independent variables.

### 2.7. Statistics

The NEMRODW software was utilized to conduct the variable study as well as to perform analyses of variance (ANOVA), regression and graphical studies.

## 3. Results and Discussion

### 3.1. Alginate Characterization

**Figure 2:**
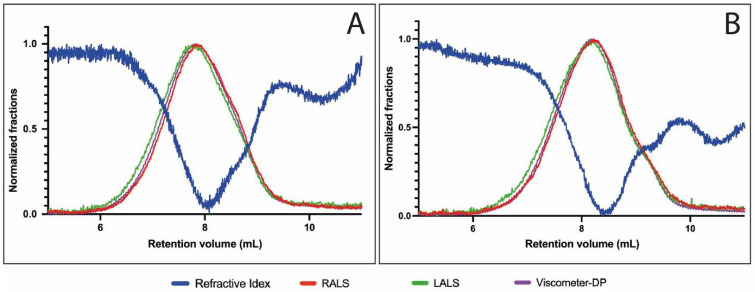
Size-exclusion chromatograms for AlgLF (**A**) and AlgP (**B**) alginates.

As observed by size-exclusion chromatography coupled to multi-detectors, AlgLF presented a higher molecular weight (Figure 2). The determination of the Mw using light scattering gave a Mw of ~2.14 × 10^6^ g·mol^−1^ for AlgLF and a Mw of ~1.22 × 10^6^ g·mol^−1^ for AlgP.

**Figure 3:**
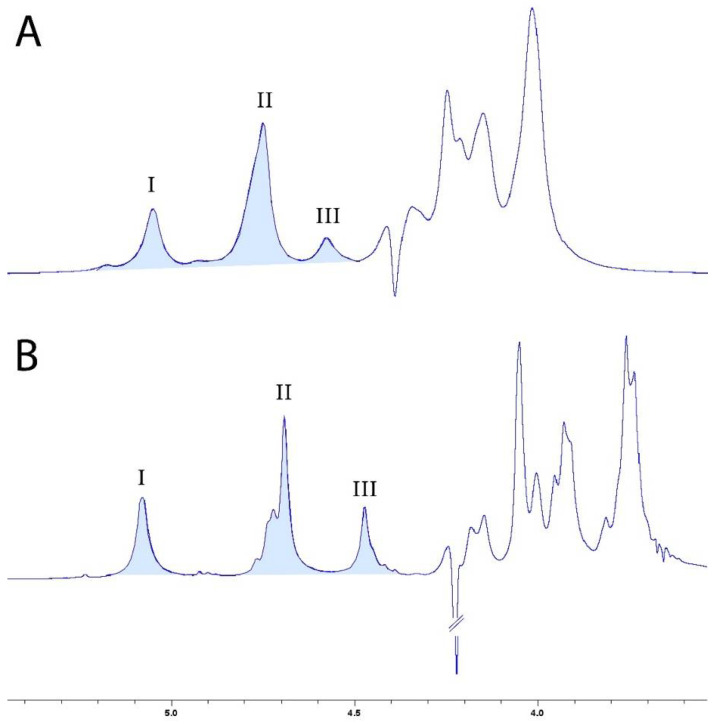
^1^H NMR spectra at 400 MHz of AlgLF (**A**) and AlgP (**B**) in D_2_O solution.

The M/G ratio is also a key characteristic that helps to determine the nature of the gel formed. A lower M/G ratio results in a brittle gel, while a higher ratio produces a more elastic gel. The ^1^H NMR spectra of the alginates are displayed in Figure 3 and reveal the presence of three peaks, characteristic of uronic acid. Peak I at 5.048 ppm and peak III at 4.446 ppm corresponded to the C1 and C5 hydrogens of guluronic acid, respectively, and peak II at 4.655 ppm corresponded to the C1 hydrogen of mannuronic acid residues.

The composition and structure of alginate-forming units were given using the methodology proposed by Grasdalen et al. [10] using the following equations:

(2) $$F_{G}=A_{I}/(A_{II}+{\;A}_{III})$$

(3) $$F_{M}=1-F_{G}$$

(4) $$F_{GG}=A_{III}/A_{II}+A_{III}$$

(5) $$F_{GM}=F_{MG}=F_{G}-F_{GG}$$

(6) $$F_{MM}=F_{MG}-F_{MG}$$

(7) $$M/G=\left(1-F_{G}\right)/F_{G}$$

where *A_I_* was the relative area of peak I; *A_II_* was the relative area of peak II; and *A_III_* was the relative area of peak III.

The distributions and M/G ratios of the alginate samples are presented in Table 3. The AlgLF and AlgP M/G ratios were determined as 2.35 and 1.72, respectively. Thus, AlgP presented higher G unit content than AlgLF.

### 3.2. Influence of Process Variables on AlgLF Beads

**Figure 4:**
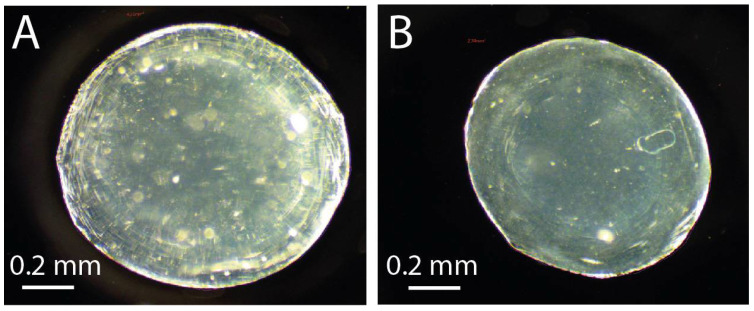
Microphotography of AlgP (**A**) and AlgLF (**B**).

In order to study the impact of the process parameters (i.e., flow rates, frequency, [CaCl_2_] and solution aging) on the alginate (AlgLF) bead size, 34 experiments were carried out, and the AlgLF beads presented an average area of 2.893 mm^2^. The microphotographs of the resulting beads are displayed in Figure 4.

The validity of the experimental design response was confirmed by the results analysis. The coefficient of determination was used to express the model consistency deduced from the Doehlert experimental design. The model presented an R^2^ = 0.852. The significance and adequacy were confirmed by analysis of variance (ANOVA) (Table 4).

Based on the results, a mathematical model was developed to predict the AlgLF bead size, as follows:

(8) $$\begin{array}{cc} Y= & 2.893+0.523x_{1}+0.788x_{2}-0.338x_{3}-2.330x_{4}-0.098x_{5}+0.317x_{1}^{2}-0.046x_{2}^{2}+0.128x_{3}^{2}+1.358x_{4}^{2}\\ & -0.290x_{5}^{2}-0.398x_{1}x_{2}+0.165x_{1}x_{3}-0.524x_{2}x_{3}-1.402x_{1}x_{4}-1.363x_{2}x_{4}+0.494x_{3}x_{4}\\ & -0.932x_{1}x_{5}-0.856x_{2}x_{5}-0.158x_{3}x_{5}+0.799x_{4}x_{5} \end{array}$$

From the general Equation (8), to represent the impact of each factor’s individual evolution for each variable, the expressions can be presented as follows:

(9) $$f_{1}=0.523x_{1}+0.317x_{1}^{2}$$

(10) $$f_{2}=0.788x_{2}-0.046x_{2}^{2}$$

(11) $$f_{3}=-0.338x_{3}+0.128x_{3}^{2}$$

(12) $$f_{4}=-2.330x_{4}+1.358x_{4}^{2}$$

(13) $$f_{5}=-0.098x_{5}-0.290x_{5}^{2}$$

**Figure 5:**
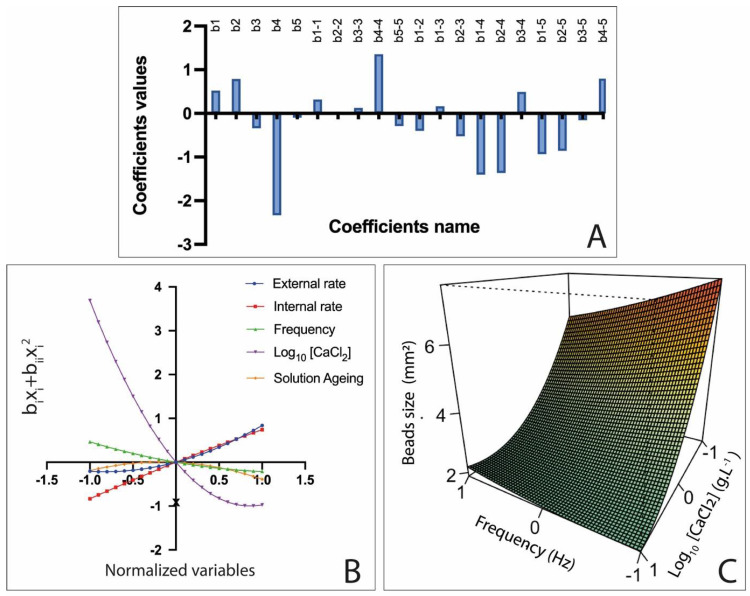
Pareto chart of main effects obtained from AlgLF Doehlert experimental design (**A**); regression of f_1_, f_2_, f_3_, f_4_ and f_5_ as function of factor levels (**B**); surface plots showing effects of mutual interaction between frequency and calcium chloride concentration (log_10_[CaCl_2_]) on AlgLF bead size (**C**), while the other variables were kept at their center points.

Using Equations (9)–(13), a graphical regression was realized and is presented in Figure 5. Therefore, the impacts of the variables were realized individually.

It appeared that the highest coefficient value corresponded to b_4_ and b_4-4_, which indicated that the log_10_ [CaCl_2_] had the greatest individual impact on the bead size, as illustrated in Figure 5A. Therefore, from the individual graphical regression of Equation (12), a decrease in the alginate bead size was observed with the increase in the calcium ion concentration, as illustrated in Figure 5B.

This behavior has been previously associated with alginate network tightening [18,19]. Further, coefficients b_1-4_ and b_2-4_ corresponding to alginate rates and log_10_ [CaCl_2_] interactions corroborated the antagonistic effect between the alginate and the calcium ion concentration. Additionally, the X_3_ factor exhibited an antagonistic effect, as evidenced by coefficient b_3_ (−0.338). Increasing the frequency resulted in a bead size reduction, as the fluid flow was more swiftly interrupted at each vibration break, causing the solution jet to break more frequently [20]. Solution aging seemed to have a minimal and non-significative impact on the bead size; more statistical data are provided in Table 5.

### 3.3. Influence of Variables on AlgP Beads

The effects of the experimental conditions on the AlgP bead size were also modeled. The experimental data were well adjusted by the generated model, as expressed by the coefficient of determination (R^2^ = 0.935). Further, statistical significance was determined through ANOVA and showed a *p*-value < 0.01, indicating that the model significance levels were in the range between 99% and 99.9% (Table 6). AlgP beads presented an average area of 3.133 mm^2^.

The AlgP bead size response was modeled with the following quadratic Equation (14):

(14) $$\begin{array}{cc} Z= & 3.133+0.848x_{1}+0.758x_{2}-0.480x_{3}-1.914x_{4}+0.253x_{5}-0.098x_{1}^{2}+0.306x_{2}^{2}-0.253x_{3}^{2}+1.199x_{4}^{2}\\ & +0.557x_{5}^{2}+0.306x_{1}x_{2}-0.451x_{1}x_{3}+0.638x_{2}x_{3}-0.855x_{1}x_{4}-0.375x_{2}x_{4}+0.747x_{3}x_{4}\\ & +0.522x_{1}x_{5}-1.018x_{2}x_{5}-1.261x_{3}x_{5}-0.100x_{4}x_{5} \end{array}$$

To represent the impact of each factor’s individual progression, each variable was studied through individual equations adapted from Equation (14) as follows:

(15) $$f_{1}=0.848x_{1}-0.098x_{1}^{2}$$

(16) $$f_{2}=0.758x_{2}+0.306x_{2}^{2}$$

(17) $$f_{3}=-0.480x_{3}-0.253x_{3}^{2}$$

(18) $$f_{4}=-1.914x_{4}+1.199x_{4}^{2}$$

(19) $$f_{5}=0.253x_{5}+0.557x_{5}^{2}$$

**Figure 6:**
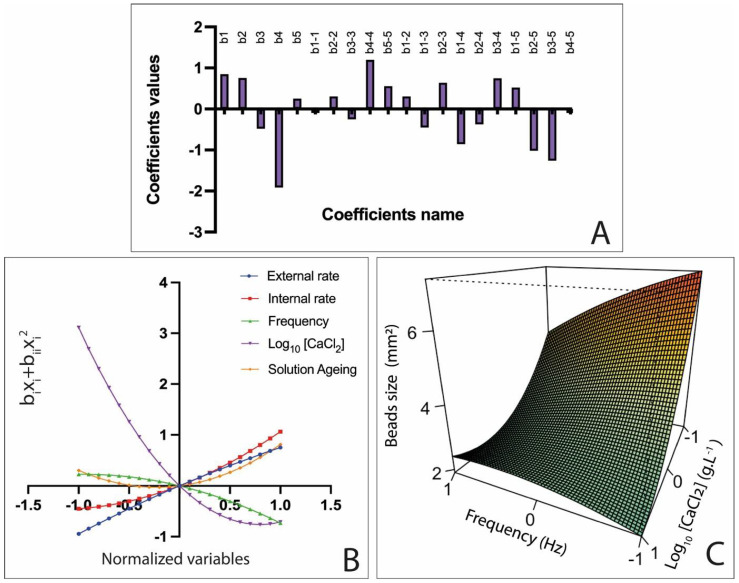
Pareto chart of main effects obtained from AlgP Doehlert experimental design (**A**); regression of f_1_, f_2_, f_3_, f_4_ and f_5_ as function of factor levels (**B**); surface plots showing effects of mutual interaction between frequency and calcium chloride concentration (log_10_[CaCl_2_]) on AlgP bead size (**C**), while the other variables were kept at their center points.

Using Equations (15)–(19), a graphical regression was realized and is presented in Figure 6. Therefore, the impacts of the variables were realized individually.

Based on Equation (14), describing the AlgP bead size, the coefficients with the highest value were b4 and b_4-4_, signifying that here, also, the log_10_ [CaCl_2_] had a significative negative impact on the AlgP bead size. Consequently, as reported previously, an increase in the calcium ion concentration led to a decrease in the alginate bead size. Furthermore, coefficients b_1-4_ and b_2-4_, representing interactions between the AlgP solution rates and log_10_ [CaCl_2_], confirmed the antagonistic effect between the alginate and the calcium ion concentration.

Overall, as for AlgLF beads, the AlgP bead size was mostly impacted by the calcium ion concentration, alginate solution rate and applied frequency, although the impact of solution aging on the bead size appeared to be minimal and statistically insignificant (Table 5).

### 3.4. Alginate Characteristics’ Impact on Gelation

Considering the experimental design results concerning the influence of the log_10_ [CaCl_2_] on both batches, calcium chloride acted as a cross-linking agent, interacting mostly with the G-blocks of alginate molecules to produce gel networks. Elevated concentrations of calcium chloride facilitated enhanced cross-linking and engendered robust gels. Moreover, alginate gelation was influenced by the proportion of G (guluronic acid) and M (mannuronic acid) units, which affected the rotation around the glycosidic linkage [21].

A mathematical phenomenological model was used to study the beads’ reticulation and observe the two-phase gelling phenomenon previously established [19]. The application of Equations (8) and (14) for various values of Q/F and log_10_ [CaCl_2_] were used to determine the final volume of beads from the calculated area vs. alginate flow for both AlgLF and AlgP beads, respectively. Based on the experimental design results and on this phenomenological model, the alginate rate and calcium ion concentration’s impacts on bead size variation can be described as follows:

(20) $$\Delta V=\left(\frac{Q}{F}\right)C$$

where ΔV = bead volume variation (V-V0); Q = total flow rate; F = frequency; C = coefficient of size variation—if C = 0, no size variation is observed; if C > 0, volume expansion is observed; and if C < 0, alginate retraction and/or erosion occurs.

**Figure 7:**
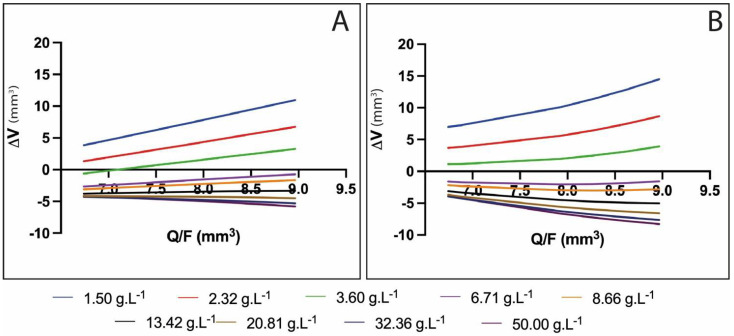
Mathematically predicted values for AlgP (**A**) and AlgLF (**B**) bead volume variations versus Q/F for determination of C coefficients (draw slopes) using nine CaCl_2_ concentrations from 1.5 to 50 g·L^−1^, while other variables were kept at 120 Hz for frequency and 47 h for solution aging.

A clear relationship was observed between the bead volume and alginate flow rate, showing a linear evolution (Figure 7).

As shown in Figure 7, ΔV traduced the volume change between the drop volume before entering the CaCl_2_ solutions, V0, and the final volume of reticulated beads, V. Depending on the CaCl_2_ concentration, three reticulation behaviors were observed.

1. For the lowest concentrations (below 3.6 g/m^2^), ΔV ≥ 0 was measured for all flow rates. This means that no significant shrinking occurred when the drop penetrated into the calcium solution. Due to the low ionic gradient between the internal water and calcium solution, no significant water release was observed and low and light reticulation occurred. In all cases, the slopes of the draws were positive (C > 0).
2. For CaCl_2_ concentrations between 6.71 and 13.42 g/m^2^, significant water release and shrinking was observed, leading to V < 0 for all Q/F values. Nevertheless, the bead volume increased with (Q/F), due to the lower relative shrinkage for larger beads. The slopes of the draws were still positive (C > 0) but, as the CaCl_2_ concentration rose, the slope was reduced, corresponding to fewer beads growing with (Q/F). This means that a denser network was formed as more Ca^2+^ ions penetrated the alginate (enhancing reticulation, contracting the alginate network, and reducing the bead size).
3. For excessive CaCl_2_ concentrations (above 13.42 g/m^2^), Ca^2+^ supersaturation occurred, leading to repulsion between alginate chains, the large release of water from the capsule and free alginate dissolution into the surrounding solution (erosion).

**Figure 8:**
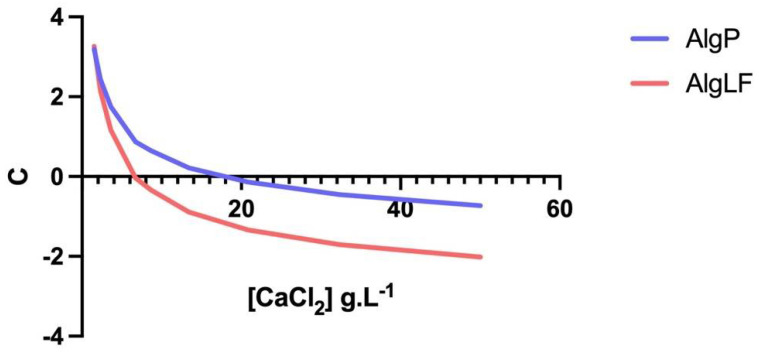
C coefficient versus CaCl_2_ concentration for C coefficient modeling.

Moreover, the non-linear evolution of ΔV was observed for AlgLF, which could be due to the M/G ratio. The higher M content conferred more elasticity to the alginate network, and therefore resulted in a less organized network during gelation. In Figure 7, the slope of each curve corresponds to the C values (Equation (20)). The C coefficient changes vs. the CaCl_2_ concentration are presented in Figure 8, for both AlgLF and AlgP. The C values were initially positive and then became negative for both alginates. A C sign change occurred at high concentrations for AlgP compared to AlgLF. The AlgP curve was always above the AlgLF curve.

Due to the lower M/G ratio (1.72) and higher molecular weight, AlgP produced larger beads than AlgLF. As AlgP contained a higher proportion of G-blocks, increasing the calcium chloride concentration resulted in stronger and more extensive cross-linking. As a result, bead formation presented reduced syneresis, leading to larger bead sizes.

The principal difference between high- and low-molecular-weight alginates in their binding behavior manifested primarily in the final stage of reticulation, as described by Fang et al. (2007) in three steps via isothermal titration calorimetry. The steps were distinguished by Ca/G-block ratios as monocomplex formation, pairing into egg-box dimers and lateral association. In the case of high-molecular-weight alginates coupled with lower G-block content (AlgLF), this resulted in smaller beads.

**Figure 9:**
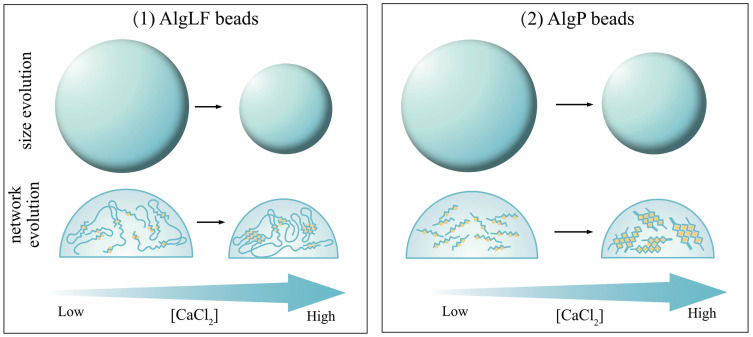
Illustration of the [CaCl_2_] impact on alginate (AlgLF and AlgP presenting high Mw/low G-blocks and low Mw/high G-blocks, respectively) network during bead formation mechanism with constant alginate amount. Adapted from [19].

In the present research, the mannuronate (M) and guluronate (G) content impacted the alginate backbone. The M-block conformation resulted in less compact alginate chains. Thus, glycosidic linkage and rotation was favored, leading to a volume effect between chains [22]. The volume variation capabilities increased during gelation, leading to a higher tightening coefficient. Alginates’ characteristics impacted the alginate network during gelation, as presented in Figure 9. These observations were in line with those made on alginate-based core–shell capsules.

## 4. Conclusions

This study investigated bead production using two types of alginates, AlgLF and AlgP, through electro-vibrating extrusion. The results demonstrated that the calcium chloride concentration had the most significant impact on the bead size, with a higher concentration leading to smaller beads [23].

The phenomenological model allowed the examination of alginate reticulation in bead form versus core–shell capsules. It was observed that AlgP, with a lower M/G ratio and a higher proportion of G-blocks, exhibited an extended gelation process, stronger cross-linking and larger bead sizes.

**Figure 10:**
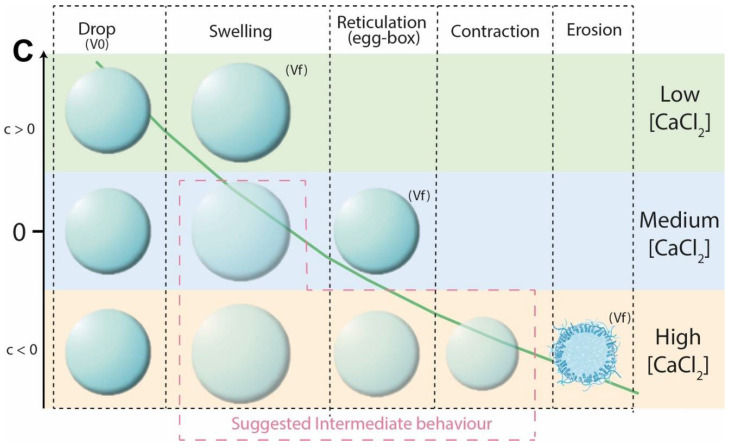
Scheme illustrating size variation and alginate network formation in low-, medium- and high-concentration gelling baths.

The M/G ratio and content of mannuronate and guluronate impacted the alginate network, affecting the glycosidic linkage and rotation between chains during gelation. This led to a higher tightening coefficient and increased volume variation capabilities in AlgLF compared to AlgP. Furthermore, the study linked the alginate bead size to previous microscopic observations (as illustrated in Figure 10) related to alginate binding, with the formation of monocomplexes and egg-box dimers and the lateral association of egg-box dimers [24]. The differences in the molecular size and intra-cluster association of egg-box dimers between high- and low-molecular-weight alginates explained the variations in bead size for AlgLF and AlgP.

In summary, the findings of this study highlight key factors influencing bead size in alginate production, namely the calcium chloride concentration, the alginate molecular weight and the M/G ratio. This knowledge could be valuable in optimizing the fabrication of alginate-based beads for various applications, including foods, cosmetics and other biomedical and pharmaceutical uses.

## Figures and Tables

**Table 1:** Levels of independent variables used in experimental design.

Factor	Name	Unit	Low-Level Value	High-Level Value	Number of Levels
X1	External flow rate	mL·min^−1^	30	50	5
X2	Internal flow rate	mL·min^−1^	4	18	7
X3	Frequency	Hz	80	200	7
X4	Log_10_ [CaCl_2_]	g·L^−1^	0.176	1.699	7
X5	Solution aging	Hours	24	72	3

**Table 2:** Calcium concentrations corresponding to log_10_ [CaCl_2_] variable.

Log_10_ [CaCl_2_]	[CaCl_2_] g·L^−1^
0.176	1.50
0.328	2.12
0.785	6.09
0.937	8.66
1.089	12.29
1.546	35.21
1.699	50.00

**Table 3:** ^1^H NMR analysis of alginate samples.

Sample	Composition, Fraction		Doublet Frequencies				M/G
	F_G_	F_M_	F_GG_	F_GM_	F_MM_	F_MG_	
AlgLF	0.30	0.70	0.11	0.18	0.52	0.18	2.35
AlgP	0.37	0.63	0.29	0.07	0.56	0.07	1.72

**Table 4:** ANOVA analysis of AlgLF bead size response from Doehlert experimental design.

Response	Source of Variation	Sum of Squares	Degree of Freedom	Mean Square	F-Value	Significance
Bead size (Area)	Regression	47.9642	20	2.3982	3.735	0.920 **
	Residual	8.3476	13	0.6421		
	Lack-of-fit	6.2833	10	0.6283	0.913	6.04
	Pure error	2.0643	3	0.6881		
	Total	56.3118	33			

(**): significant at the levels comprised between 99% and 99.9% (for 0.001 < *p* value < 0.01).

**Table 5:** Model coefficient analysis of Doehlert design for AlgLF and AlgP bead size.

	Coefficient		Standard Error		t-Value		Significance	
	AlgLF	AlgP	AlgLF	AlgP	AlgLF	AlgP	AlgLF	AlgP
b0	2.893	3.133	0.401	0.227	7.22	13.81	<0.01 ***	<0.01 ***
b1	0.523	0.848	0.327	0.185	1.60	4.58	13.4	0.0514 ***
b2	0.788	0.758	0.327	0.185	2.41	4.10	3.16 *	0.126 **
b3	−0.338	−0.480	0.327	0.185	−1.03	−2.59	32.1	2.22 *
b4	−2.330	−1.914	0.327	0.185	−7.12	−10.34	<0.01 ***	<0.01 ***
b5	−0.098	0.253	0.327	0.185	−0.30	1.37	76.9	19.5
b1-1	0.317	−0.098	0.694	0.393	0.46	−0.25	65.5	80.8
b2-2	−0.046	0.306	0.694	0.393	−0.07	0.78	94.8	45.0
b3-3	0.128	−0.253	0.654	0.370	0.20	−0.68	84.8	50.6
b4-4	1.358	1.199	0.621	0.351	2.19	3.41	4.75 *	0.462 **
b5-5	−0.290	0.557	0.594	0.336	−0.49	1.66	63.3	12.2
b1-2	−0.398	0.306	0.925	0.524	−0.43	0.58	67.4	56.9
b1-3	0.165	−0.451	1.035	0.585	0.16	−0.77	87.5	45.5
b2-3	−0.524	0.638	1.034	0.585	−0.51	1.09	62.1	29.6
b1-4	−1.402	−0.855	1.075	0.608	−1.30	−1.41	21.5	18.3
b2-4	−1.363	−0.375	1.075	0.608	−1.27	−0.62	22.7	54.8
b3-4	0.494	0.747	1.075	0.608	0.46	1.23	65.3	24.1
b1-5	−0.932	0.522	1.095	0.620	−0.85	0.84	41.0	41.5
b2-5	−0.856	−1.018	1.095	0.620	−0.78	−1.64	44.8	12.4
b3-5	−0.158	−1.261	1.095	0.620	−0.14	−2.03	88.8	6.3
b4-5	0.799	−0.100	1.095	0.620	0.73	−0.16	47.8	87.5

(***): significant at the level > 99.9% (for 0.0001 < *p*-value < 0.001). (**): significant at the levels comprised between 99% and 99.9% (for 0.001 < *p*-value < 0.01). (*): significant at the levels comprised between 95% and 99% (for 0.01 < *p*-value < 0.05).

**Table 6:** ANOVA analysis of AlgP bead size response from Doehlert experimental design.

Response	Source of Variation	Sum of Squares	Degree of Freedom	Mean Square	F-Value	Significance
Bead size (Area)	Regression	38.2615	20	1.9131	9.301	<0.01 ***
	Residual	2.6738	13	0.2057		
	Lack-of-fit	2.5397	10	0.2540	5.683	9.0
	Pure error	0.1341	3	0.0447		
	Total	40.9352	33			

(***): significant at the level > 99.9% (for 0.0001 < *p*-value < 0.001.

## Data Availability

The data presented in this study are available on request from the corresponding author.
